# Prevalence of Self-Reported Gluten Sensitivity and Adherence to a Gluten-Free Diet in Argentinian Adult Population

**DOI:** 10.3390/nu9010081

**Published:** 2017-01-21

**Authors:** Francisco Cabrera-Chávez, Gimena V. A. Dezar, Anna P. Islas-Zamorano, Jesús G. Espinoza-Alderete, Marcela J. Vergara-Jiménez, Dalia Magaña-Ordorica, Noé Ontiveros

**Affiliations:** 1 Nutrition Sciences Academic Unit, Universidad Autónoma de Sinaloa, Culiacán, Sinaloa 80019, Mexico; fcabrera@uas.edu.mx (F.C.-C.); islasap@hotmail.com (A.P.I.-Z.); jesus.93106@gmail.com (J.G.E.-A.); mjvergara@uas.edu.mx (M.J.V.-J.); dmagana@uas.edu.mx (D.M.-O.); 2 Facultad de Bioquímica y Ciencias Biológicas, Universidad Nacional del Litoral, Santa Fe 3000, Argentina; gdezar@unl.edu.ar; 3 Regional Program for PhD in Biotechnology, FCQB, Universidad Autónoma de Sinaloa, Culiacán, Sinaloa 80019, Mexico

**Keywords:** gluten, non-celiac gluten sensitivity, non-celiac wheat sensitivity, wheat allergy, celiac disease, prevalence

## Abstract

Background: Previous studies suggest that the prevalence of wheat/gluten sensitivity and adherence to a gluten-free diet (GFD) are high in Latin population despite a poor diagnosis of celiac disease. However, these prevalence rates still remain unknown in most Latin American countries. Methods: A cross-sectional survey study was conducted in Santa Fe, Argentina. Results: The estimated self-reported prevalence rates were (95% Confidence Interval [CI]): self-reported gluten sensitivity (SR-GS) 7.61% (6.2–9.2), SR-GS currently following a GFD 1.82% (1.2–2.7), celiac disease 0.58% (0.3–1.2), wheat allergy 0.33% (0.12–0.84), self-reported non-celiac gluten sensitivity (SR-NCGS) 6.28% (5.1–7.8), SR-NCGS currently following a GFD 0.91% (0.5–1.6), and adherence to a GFD 6.37% (5.1–7.9). SR-GS was more common in women (6.0%; *p* < 0.001) and associated with irritable bowel syndrome (*p* < 0.001). Among the GFD followers, 71.4% were doing it for reasons other than health-related benefits and 50.6% without medical/dietitian advice. In the non-SR-GS group, the main motivations for following a GFD were weight control and the perception that a GFD is healthier. Conclusion: In Argentina, gluten sensitivity is commonly reported and it seems that physicians/gastroenterologists are aware of celiac disease diagnosis. Trustable information about the benefits and potential consequences of following a GFD should be given to the general population.

## 1. Introduction

The term “gluten-related disorders” encompasses all conditions related to gluten intake; it includes autoimmune, allergic, and non-autoimmune and non-allergic diseases [1,2]. Celiac disease is an autoimmune-like gluten-related disorder, which is triggered by gluten from wheat, rye and barley. This condition affects between 0.5% and 1% of the general population [3]. Wheat allergy is a condition that can be mediated or not by allergen-specific IgE antibodies and its prevalence in adult population is unknown in many countries [4]. A third gluten-related disorder is non-celiac gluten sensitivity (NCGS). These are cases where celiac disease and wheat allergy have been ruled-out, but symptomatic relief is reached after gluten withdrawal and a symptomatic relapse is confirmed upon reintroduction of gluten-containing food [5]. Because NCGS manifestations could be triggered by wheat components other than gluten, such as low-fermentable, poorly-absorbed, short-chain carbohydrates (FODMAPs) [6] and the wheat components amylase trypsin inhibitors [7], the abbreviation NCGS is evolving to non-celiac wheat sensitivity (NCWS) [8]. Experts have proposed diagnostic criteria for NCGS [9], but there is no a well-accepted gold standard for this purpose yet [8]. This and the lack of biological markers to support the diagnosis of NCGS make it difficult to estimate the population prevalence of this disorder. Alternatively, the self-reported gluten sensitivity (SR-GS) and/or self-reported NCGS (SR-NCGS) prevalence rates have been estimated. This prevalence rates varies among populations and fluctuates between 0.5% and 13% [10,11,12,13].

Following a gluten-free diet (GFD) is the only accepted treatment for gluten-related disorders. Patients following a GFD should be instructed by a trained physician/dietitian in order to avoid micronutrients deficiencies [14,15,16] and improve fiber intake [15]. Although the diet is considered a treatment, it seems that most people following a GFD are doing it for reasons other than health-related benefits and probably without medical/dietitian advice [11,12]. Studies addressing both the motivations for following a GFD without a physician-diagnosed gluten-related disorder and who instructs the diet in this group of people are scarce. The aim of the present study was to estimate the prevalence rates of SR-GS, SR-NCGS, and self-reported wheat allergy in adult population from Santa Fe, Argentina. The prevalence of adherence to a GFD as well as the motivations for adhering to the diet and who instructs the GFD were aspects also investigated.

## 2. Materials and Methods

### 2.1. Population Survey

We conducted a self-administered questionnaire-based cross-sectional study in Santa Fe, Argentina. All data were collected during the period from August to September 2016. The survey was conducted as previously described [11,12]. Briefly, respondents were approached in urban parks and outside shopping malls and supermarkets located in Santa Fe city. Inclusion criteria were: (1) Argentinian individuals; (2) ≥18 years old; and (3) subjects being able to read and answer the questionnaire by themselves. Assistance on specific terms was given when it was requested.

### 2.2. Questionnaire

A previously validated Spanish version of a self-administered questionnaire was utilized for the study purposes [11]. The questionnaire has two sections. The first section was designed for those who reported adverse reactions to oral wheat and/or gluten. The second section was designed for those who reported adverse reactions to foods other than wheat/gluten or reported no adverse reactions to foods [11,12]. The questionnaire was slightly modified to inquire about the motivations for following a GFD and who instructs the diet (Supplementary Materials Section S1).

### 2.3. Definitions

Adverse reactions to food were considered when the respondents reported that the food-induced symptoms occurred always or most of the time (recurrent) or sometimes (non-recurrent) [11,12]. Self-reported physician-diagnosed celiac disease or wheat allergy was considered when the respondents reported that a physician diagnosed them and were also following a GFD [11,12,17]. Additionally, self-reported wheat allergy was considered when the respondents reported recurrent adverse reactions convincing of food allergy and were also following a GFD, as previously described [11,12,18]. SR-GS was considered when the respondents met criteria for recurrent adverse reactions to oral wheat/gluten. Self-reported physician-diagnosed NCGS was considered when the respondents reported that a physician diagnosed them. SR-NCGS was considered when the respondents met the following: (1) respondents who did not meet criteria for self-reported physician-diagnosed celiac disease or wheat allergy; (2) respondents who did not meet criteria for self-reported wheat allergy; and (3) respondents who met criteria for recurrent adverse reactions to wheat/gluten (SR-GS) [12].

### 2.4. Statistical and Ethical Issues

Statistical analysis was carried out using PASW statistics version 18.0 (SPSS Inc., Chicago, IL, USA). Categorical variables were summarized by descriptive statistics, including total numbers, percentages, odds ratio, and 95% confidence interval (CI). Associations were analyzed by two-tailed Fisher’s exact test. Continuous variables were summarized by mean and range with differences between groups calculated using the Student *t*-test. A *p* value < 0.05 was considered statistically significant. Prevalence rates were calculated using OpenEpi software version 3.03a [19]. Rates were reported as rate (95% CI) per 100 inhabitants. All respondents gave informed consent in writing to participate in the study. Ethics Review Board of the Universidad Nacional del Litoral approved the protocol. Ethical approval number Acta 09/16.

## 3. Results

### 3.1. Study Participants and Demographic Characteristics

A total of 1209 individuals completed the questionnaire in its entirety. The response rate was 53.3%. The mean age in years was 30 (range: 18–84) and the proportion of women was slightly higher than men (52.44%) (*p* > 0.05). The demographics and clinical characteristics of the studied population are given in Table 1. Non-food allergy and colitis were more common in men than in women (*p* < 0.05). Eating disorders were more common in women (*p* < 0.05). There were no significant differences by gender for the other self-reported physician-diagnosed conditions.

### 3.2. Estimated Prevalence Rates

**Figure 1:**
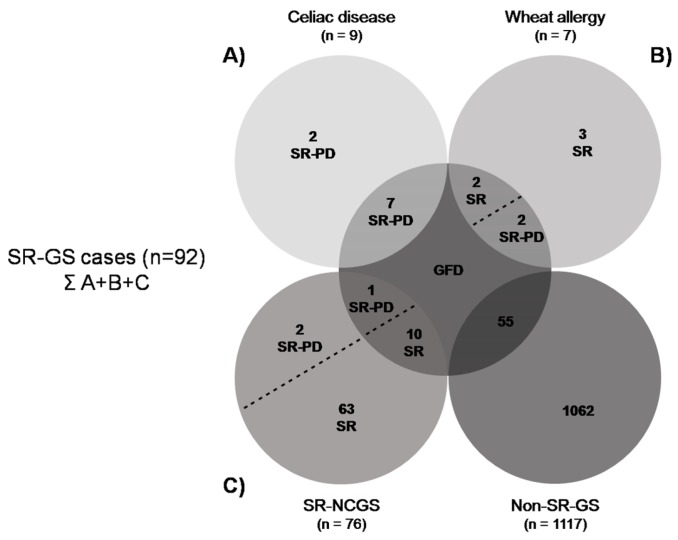
Characteristics of respondents following a GFD. SR: self-reported; SR-NCGS: self-reported non-celiac gluten sensitivity; Non-SR-GS: non self-reported gluten sensitivity; SR-PD: self-reported physician-diagnosed. (**A**–**C**) SR-celiac disease, SR-wheat allergy and SR-NCGS cases either following a GFD or not. Only those cases that reported adherence to a GFD were considered for prevalence rates estimations.

**Figure 2:**
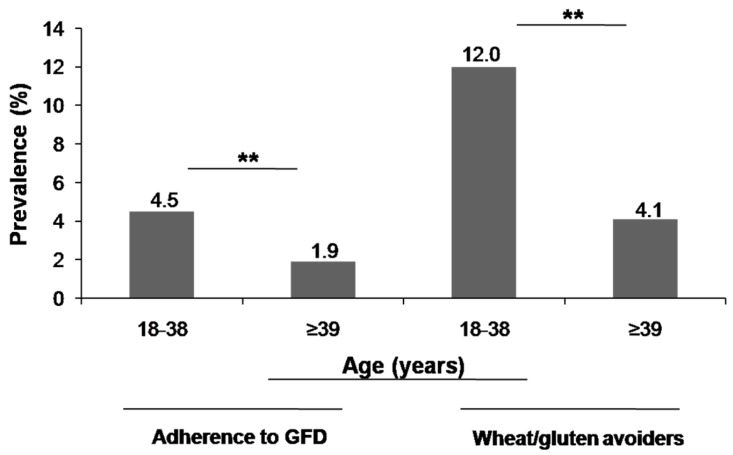
Prevalence of adherence to a GFD and avoidance of wheat/gluten-containing foods stratified by age (years). ** *p* < 0.01.

Prevalence rates estimations are shown in Table 2. Adverse reactions to food, either recurrent or not, and SR-NCGS prevalence rates were significantly higher in women than in men (*p* < 0.001). The prevalence of SR-GS following a GFD was 1.82% (*n* = 22) (95% CI 1.2–2.7). Consequently, only 28.57% (22 out of 77) of the respondents following a GFD were doing it for health-related benefits. The characteristics of the respondents who reported current adherence to a GFD are shown in Figure 1. Previous studies estimated the prevalence rates of SR-NCGS based on GFD adherence and exclusion of celiac disease [20]. Under these criteria, the estimated prevalence rate of SR-NCGS in Argentinian population was 5.78% (*n* = 70) (95% CI 4.6–7.2). However, excluding the non-SR-GS respondents who reported current adherence to a GFD (*n* = 55) and those who met criteria for self-reported wheat allergy ((*n* = 4); prevalence rate 0.33%; 95% CI 0.12–0.84), the prevalence rate of SR-NCGS currently following a GFD was 0.91% (*n* = 11) (95% CI 0.5–1.6). The prevalence of gluten avoiders (not following a GFD) was 16.13% (*n* = 195) (95% CI 14.2–18.3), but only 22.56% (*n* = 44) of them met criteria for SR-GS. The prevalence rates of adherence to a GFD or wheat/gluten avoidance were significantly higher among respondents <39 years old than those ≥39 years old (*p* < 0.01) (Figure 2). Stratified by gender, more women than men reported current adherence to a GFD (*p* > 0.05) or reported current avoidance of wheat/gluten-containing foods (*p* < 0.01).

### 3.3. Characteristics of Subjects with SR-GS and Non-SR-GS

The characteristics of SR-GS and non-SR-GS respondents are shown in Table 3. Comparisons between these two groups showed significant differences for IBS (*p* < 0.001), eating disorders (*p* < 0.05) and lactose intolerance (*p* < 0.01). These self-reported physician-diagnosed diseases were more common in SR-GS than in non-SR-GS cases (Table 3). Statistical comparisons between SR-GS and those who reported recurrent adverse reactions to foods other than wheat/gluten were not significant for any of the self-reported physician-diagnosed diseases assessed (*p* > 0.05) (Supplementary Materials Table S1).

### 3.4. Recurrent Symptoms Related to Wheat/Gluten Ingestion

**Figure 3:**
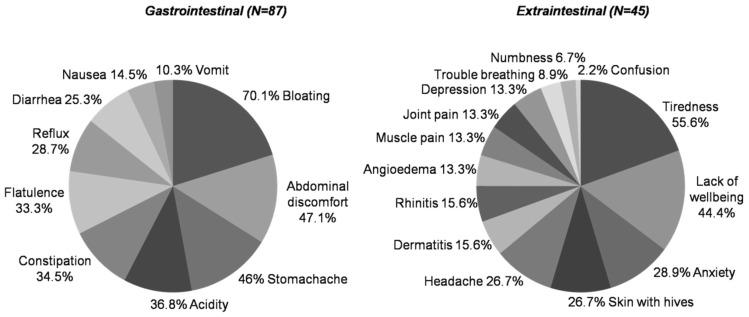
Recurrent self-reported symptoms in SR-GS cases.

Gastrointestinal symptoms were reported for 87 out of 92 SR-GS cases. The most commonly reported gastrointestinal symptoms were bloating (*n* = 61; 70.1%), abdominal discomfort (*n* = 41; 47.1%), and stomachache (*n* = 40; 46.0%) (Figure 3). Extraintestinal symptoms were reported for 45 SR-GS cases. The most common extraintestinal symptoms were tiredness (*n* = 25; 55.6%), lack of wellbeing (*n* = 20; 44.4%), and anxiety (*n* = 13; 28.9%) (Figure 3). These symptoms, either gastrointestinal or extraintestinal, were also the most common manifestations in SR-NCGS cases (Supplementary Materials Figure S1). Bloating and stomachache were the most common symptoms in those who reported adverse reactions to foods other than gluten (Supplementary Materials Figure S2). Comparisons between the SR-GS and self-reported recurrent adverse reactions to foods other than gluten groups showed significant differences for bloating and constipation (*p* < 0.05) (Supplementary Materials Table S2).

### 3.5. Motivations for Following a GFD or for Avoiding Wheat/Gluten and who Instructs the GFD

**Figure 4:**
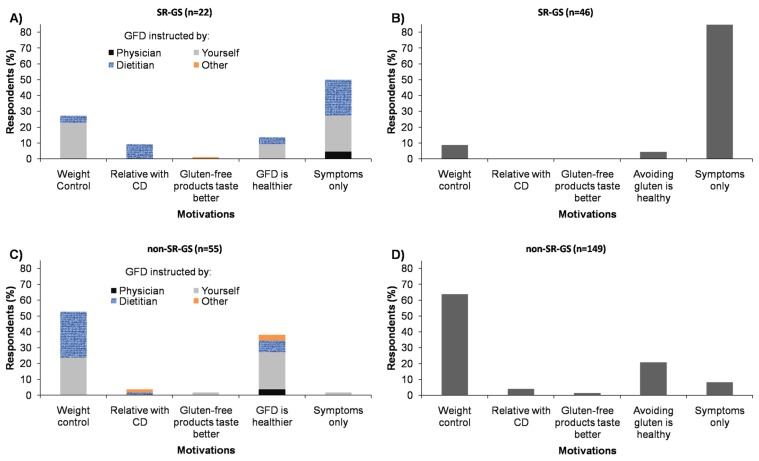
Motivations for following a GFD or avoiding wheat/gluten-containing foods and who instructs the GFD: (**A**) SR-GS individuals currently following a GFD; and (**B**) SR-GS individuals currently avoiding wheat/gluten-containing foods. In Figure 4A,B, in addition to the symptoms triggered by gluten intake, 11 and 6 individuals reported other motivations for following a GFD or avoiding wheat/gluten-containing foods, respectively; (**C**) Non-SR-GS individuals currently following a GFD; and (**D**) non-SR-GS individuals currently avoiding wheat/gluten-containing foods. In Figure 4C,D, 1 and 12 individuals reported non-recurrent adverse reactions to gluten intake as the motivation for following a GFD.

In addition to the recurrent symptoms triggered by gluten intake, other motivations for following a GFD or for avoiding wheat/gluten from the diet were weight control and the perception that a GFD is healthier or avoiding wheat/gluten is healthy (Figure 4A,B). These motivations were also reported by most non-SR-GS cases that were following a GFD or were avoiding wheat/gluten from their diets (Figure 4C,D). Comparisons between the SR-GS and non-SR-GS groups currently following a GFD showed significant associations for weight control (*p* < 0.05) and the perception that a GFD is healthier (*p* < 0.05), these motivations were more commonly reported by the non-SR-GS group (Supplementary Materials Table S3). Regarding who instructs the GFD, 14 out of 22 (63.6%) SR-GS and 24 out of 55 (43.6%) non-SR-GS cases reported that they were seeing a physician/dietitian for gluten-free dietary advice (*p* > 0.05) (Figure 4A,C). Thus, among those who reported current adherence to a GFD (*n* = 77), 50.65% (*n* = 39) were doing it without medical/dietitian advice (Figure 4A,C).

Next, we stratified by gender the motivations for following a GFD and who instructs the diet. In the SR-GS group (15 women and seven men currently following a GFD), 50% (*n* = 11; seven women and four men) of the cases reported the symptoms triggered after wheat/gluten intake as the only reason for following a GFD (Supplementary Materials Table S4). Regarding who instructs the GFD, 45.45% (*n* = 10; seven women and three men) of the respondents from the SR-GS group reported that were seeing a physician/dietitian for gluten-free dietary advice (Supplementary Materials Table S4). In the non-SR-GS group (29 women and 26 men following a GFD), more women than men (*n* = 18; 62.07% vs. *n* = 11; 42.30%) reported weight control as the motivation for following a GFD (*p* > 0.05). A slightly higher proportion of men than women reported that a GFD was healthier (*n* = 11; 42.31% vs. *n* = 11; 37.93%) (*p* > 0.05). Regarding who instructs the GFD, more women than men (*n* = 17; 58.62% vs. *n* = 7; 26.92%) reported that were seeing a physician/dietitian for gluten-free dietary advice (*p* < 0.05). Consequently, more men than women (*n* = 19; 73.08% vs. *n* = 12; 41.38%) were following a GFD without medical/dietitian advice (*p* < 0.05).

## 4. Discussion

This study has shown that recurrent adverse reactions to foods are commonly attributed to wheat/gluten intake. SR-GS was informed by 7.6% of the studied population. This prevalence estimation is consistent with previous survey studies carried out in Latin American countries and The Netherlands, which have estimated SR-GS prevalence rates between 5.3% and 7.8% [11,12,13]. Substantially higher SR-GS prevalence estimations (up to 13%) have been reported in the United Kingdom [17], but there are no enough data available to provide concise explanations about these discrepancies. According to others [13], media attention is a factor that might explain such differences. Findings consistent between the present and previously published survey studies include the following: SR-GS is predominant in women; the most common symptoms reported are bloating, abdominal discomfort, abdominal pain, tiredness, lack of wellbeing, and headache; SR-GS is significantly associated with IBS when compared to the general population [11,12,13,17]. Our results also corroborate that the percentage of self-reported physician-diagnosed IBS cases is slightly higher among those who met criteria for SR-GS than those who reported recurrent adverse reactions to foods other than wheat/gluten [11,12]. This further supports the notion that a single food (wheat) has a strong association with IBS. In fact, a recent study has shown that a high proportion of patients (up to 42.4%) with IBS met criteria for SR-NCGS [21].

The population prevalence of SR-NCGS should be estimated once self-reported celiac disease and wheat allergy have been ruled out in those who met criteria for SR-GS. Under these criteria, the prevalence of SR-NCGS in the studied population was 6.28%, which is similar to the SR-NCGS prevalence rates estimated in Mexican (4.5%) and Colombian populations (6.9%) utilizing the same instrument [11,12]. The self-reported physician-diagnosed prevalence of celiac disease in the Argentinian population was 0.58%, which is similar to that reported in the United Kingdom (0.8%) [17] and Australia (0.84%) [22]. Considering a general prevalence of celiac disease between 0.5% and 1% among populations [3] and two cases that reported a physician diagnosis of celiac disease but were not following a GFD, it can be inferred that a marked underdiagnosis of celiac disease is hardly probable in Santa Fe, Argentina. In previous studies carried out in other Latin American countries, no self-reported physician-diagnosed celiac disease cases were detected in Colombia and the self-reported physician-diagnosed prevalence of this condition in Mexican population was 0.08% (1/1238). More than fifteen years ago, studies carried out in Argentina could have estimated a self-reported physician-diagnosed prevalence rate of celiac disease of 0.05% (1/2000) [23]. However, almost ten years ago and different from Mexico and Colombia, Argentina’s Ministry of Health implemented a nationwide program for the detection and control of celiac disease [24]. Furthermore, Argentinians have subsidies to help to manage the cost of the GFD once celiac disease diagnosis has been properly established [25]. These actions could increase awareness about celiac disease among healthcare professionals and the general population and, perhaps, motivate gluten sensitive people to see physicians/gastroenterologists to undergo proper assessment of celiac disease. Regarding wheat allergy, the prevalence of this disorder in the studied population was 0.33%. This prevalence rate is consistent with previous survey studies carried out in other Latin American countries [11,12] and elsewhere [4,26,27], which have reported prevalence rates between 0.24% and <1%.

Following a GFD without a known diagnosis of celiac disease has been proposed as a surrogate marker for SR-NCGS [20]. Under these criteria, the estimated SR-NCGS prevalence rate was 5.78% in the present study. Due to the inclusion of non-SR-GS cases that reported current adherence to a GFD, the main limitation of this approach is that overestimate the prevalence of SR-NCGS currently following a GFD. In fact, this prevalence rate was 0.91% in the studied population, which is closer to the NCGS prevalence rate expected in Italians (slightly higher than 1%) [28]. Furthermore, this prevalence rate is not far from that reported in U.S. population (0.548%) applying the criteria described above [20]. Although in the U.S. study the authors stated that in-person physical examinations were performed and health questionnaires were applied to the participants of the study, the characteristics of the participants who met criteria for SR-NCGS in relation to the symptoms triggered after wheat/gluten intake were not stated [20], and this could lead to misinterpretation of the results. Accordingly, we have indicated that SR-NCGS prevalence rates estimations should be interpreted with caution and special attention should be paid to study designs [11] and the given definitions of SR-GS and/or SR-NCGS.

According to previous studies, the self-reported prevalence rate of adherence to a GFD in adult population range between 1.67% and 5.9% [11,12,17,22,29]. In the present study, the self-reported prevalence rate was 6.37% being the highest self-reported prevalence rate of adherence to a GFD ever reported in population-based studies. Notably, many respondents who reported current adherence to a GFD (71.4%) or avoidance of wheat/gluten-containing foods (77.4%) were doing it for reasons other than health-related benefits. Consistent with previous studies [22,29,30], these reasons included weight control and the public perception that a GFD is healthier or avoiding wheat/gluten-containing foods is healthy. However, following a GFD in the absence of gluten-related disorders is unlikely to confer health benefits [31,32]. Furthermore, the diet could compromise some micronutrients intake [14,15,16], especially when adhering to the diet without dietary advice, and our results show that half of those who reported current adherence to a GFD (50.6%) were doing it without medical/dietitian advice.

Our results also show that a high proportion (70.6%) of the respondents who reported recurrent adverse reactions to wheat/gluten intake (SR-GS) were not following a GFD. Although our study did not address this issue, this lack of adherence to a GFD could be related to the severity of the symptoms triggered after wheat/gluten intake and/or the availability and cost of gluten-free products, as stated by others [13]. In fact, gluten-free products are more expensive than their wheat-based counterparts and have limited availability in Santa Fe, Argentina [33].

## 5. Conclusions

This is the third of a series of studies conducted to estimate the prevalence of self-reported gluten-related disorders and adherence to a GFD in Latin American countries. The present study shows that gluten sensitivity is common, predominant in women and strongly associated with IBS. The results also suggest that Argentinian physicians/gastroenterologists are aware of celiac disease diagnosis. However, most people who reported current adherence to a GFD were doing it for reasons other than health-related benefits. Furthermore, at least half of the gluten-free followers were doing it without medical/dietitian advice. Because the main motivations for following a GFD were weight control and the perception that a GFD is healthier, giving scientifically sound information to the general population about the health-related benefits of following a GFD in the absence of a proper diagnosis of gluten-related disorders seems to be urgent.

## Figures and Tables

**Table 1:** Demographics and clinical characteristics of the studied population.

Variable *	%	*n*
Gender (male/female)	47.6/52.4	575/634
Non-food allergy	9.3	113
IBS **	5.5	66
Colitis	2.2	27
Lactose intolerance	1.9	23
Psychiatric disease	1.7	20
Food intolerance	1.3	16
Food allergy	1.2	15
Eating disorders	1.2	14
Diabetes mellitus	1.0	12
Gastrointestinal cancer	0.2	2
Hashimoto’s thyroiditis	0.2	2

* Self-reported physician-diagnosed diseases were considered for analysis; ** Irritable Bowel Syndrome.

**Table 2:** Self-reported prevalence rates estimations.

Assessment	(+) Cases *	Mean age in Years (Range)	Prevalence by Gender (95% CI)	*p* Value	General Prevalence (95% CI)
Adverse reactions to food	Total = 278	33.4 (18–84)	M 6.5 (5.2–8.0) F 16.5 (14.4–18.6)	<0.001	21.4 (19.3–23.7)
	M ** = 79				
	F ** = 199				
Adverse reactions to wheat/gluten	Total = 134	33.8 (18–74)	M 2.6 (1.8–3.7) F 8.5 (7.1–10.2)	<0.001	11.1 (9.4–12.9)
	M = 31				
	F = 103				
Recurrent adverse reactions to food	Total = 193	34.3 (18–79)	M 4.1 (3.2–5.4) F 11.8 (10.1–13.8)	<0.001	15.9 (14.0–18.1)
	M = 50				
	F = 143				
*(a) Recurrent adverse reactions to wheat/gluten (SR-GS)*	Total = 92	34.2 (18–74)	M 1.6 (1.0–2.4) F 6.0 (4.8–7.5)	<0.001	7.61 (6.2–9.2)
	M = 19				
	F = 73				
*(b) Celiac disease ^¶^*	Total = 7	26.7 (19–41)	M 0.2 (0.04–0.6) F 0.4 (0.2–1.0)	0.268	0.58 (0.3–1.2)
	M = 2				
	F = 5				
*(c) Wheat allergy ^¶^*	Total = 4	52.5 (37–59)	M 0.1 (0.01–0.5) F 0.2 (0.1–0.7)	0.350	0.33 (0.1–0.8)
	M = 1				
	F = 3				
*(d) SR-NCGS*	Total = 76	34.4 (18–74)	M 1.4 (0.9–2.2) F 5.5 (4.3–6.9)	<0.001	6.28 (5.1–7.8)
	M = 15				
	F = 61				
Adherence to GFD	Total = 77	33.8 (18–78)	M 2.7 (1.9–3.8) F 3.6 (2.7–4.8)	0.231	6.37 (5.1–7.9)
	M = 33				
	F = 44				

* Positive cases for the assessment; ** M: male; F: female; ^¶^ Two out of 9 celiac disease cases and 3 out of 7 wheat allergy cases did not report adherence to a GFD.

**Table 3:** Comparison between self-reported gluten sensitivity (SR-GS) and non-self-reported gluten sensitivity (non-SR-GS).

Variable *	SR-GS (*N* = 92) ^¶^		Non-SR-GS (*N* = 1117) ^¶^		Odds Ratio (95% CI)
	%	*n*	%	*n*	
Gender (male/female)	46.2/53.8	42/49	47.7/52.3	533/584	0.9 (0.6–1.4)
IBS	14.3	13	4.7	53	3.3 (1.7–6.4)
Food intolerance	3.3	3	1.2	13	2.9 (0.8–10.4)
Allergy	11.0	10	10.5	117	1.1 (0.5–2.1)
Psychiatric disease	0	0	1.8	20	-
Gastrointestinal cancer	1.1	1	0.1	1	12.4 (0.8–199.9)
Eating disorders	4.4	4	0.9	10	5.1 (1.6–16.6)
Autoimmune disease	2.2	2	1.1	12	2.1 (0.5–9.4)
Colitis	4.4	4	2.1	23	2.2 (0.7–6.5)
Lactose intolerance	6.6	6	1.5	17	4.6 (1.8–11.9)

* Self-reported physician-diagnosed diseases were considered for analysis; ^¶^ Age comparison between SR-GS (mean: 33.5; range: 18–74) and non-SR-GS (mean: 29.6; range: 18–84) was not significant (*p* > 0.05 by Student *t*-test).

## Supplementary Materials

The following are available online at http://www.mdpi.com/2072-6643/9/1/81/s1, S1: English and Spanish versions of the questionnaire; Table S1: Comparison between SR-GS and recurrent adverse reactions to foods other than wheat/gluten groups; Figure S1: Self-reported symptoms in the SR-NCGS group; Figure S2: Recurrent self-reported symptoms in the adverse reactions to foods other than gluten group; Table S3: Symptoms comparison between identified SR-GS and recurrent adverse reactions to foods other than wheat/gluten cases. Table S2: Motivations for following a GFD and avoiding wheat/gluten-containing foods; Table S4: Motivations for following a GFD and who instructs the GFD in the SR-GS group stratified by gender.
