# Supplementary material for: Prevalence of Self-Reported Gluten Sensitivity and Adherence to a Gluten-Free Diet in Argentinian Adult Population
Source: Nutrients. 2017 Jan 21;9(1):81. doi: 10.3390/nu9010081 (PMC5295125; doi:10.3390/nu9010081)
Supplement: Supplementary file 1 [file nutrients-09-00081-s001.pdf]

Supplementary Materials: Prevalence of Self-Reported Gluten Sensitivity and Adherence to a Gluten-Free Diet in Argentinian Adult Population

Francisco Cabrera-Chávez, Gimena V. A. Dezar, Anna P. Islas-Zamorano, Jesús G. Espinoza-Alderete, Marcela J. Vergara-Jiménez, Dalia Magaña-Ordorica and Noé Ontiveros

S1. English and Spanish Versions of the Questionnaire

English Version.

Please indicate with an X the answer to each of the following questions

1. Do you have some discomfort or adverse reaction when consuming wheat products?

Yes (Go to question 2, please answer only section 1)
No (Go to question 20, please answer only section 2)

ID: Full Name: Age: ______ Date of birth: / / Female Male Level of education

Home Phone: Cel/mobile: Email:

SECTION 1

2. Have you ever been diagnosed with a disease related to the consumption of wheat or gluten?

Yes (which of the following?) No **(go to question 5)**

a) Wheat allergy
b) Dermatitis herpetiformis
c) Coeliac disease
d) Wheat or gluten ataxia
e) Sensitivity or intolerance to wheat
f) Other (Which one?)

3. Who made the diagnosis?

a) Physician or General Practitioner (GP)
b) Gastroenterologist
c) Homeopath
d) Chemist
e) Dietitian
f) Nutritionist
g) Other, who?

4. How long ago have you been diagnosed with the disease related to the consumption of wheat? (If you do not remember, please give us an estimate)

a) 3 months or less
b) 6 months or less
c) One year or less
d) 1–5 years or less
e) 6–10 years
f) Over 10 years

5. What kind of discomfort or gastrointestinal symptoms have you shown repeatedly by consuming foods with wheat or gluten?

a) Stomach pain
b) Diarrhea
c) Vomiting
d) Nausea
e) Reflux
f) Upset stomach
g) Bloating
h) Abdominal bloating
i) Constipation
j) Acidity
k) Other (Which one?)
l) None (Please go to question 9)

6. What age were you when you first realized that foods with wheat or gluten caused you the symptoms mentioned in the previous question?__________

a) I do not remember

7. When you eat food with wheat or gluten, how often gastrointestinal symptoms mentioned above occur?

a) Always
b) Most of the times
c) Occasionally

8. After eating foods with wheat or gluten, how long it takes for gastrointestinal symptoms referred above to appear?

a) 10 min or less
b) 10 min–1 h
c) 1–2 h
d) In the first 6 h
e) Between 6 and 24 h
f) After 24 h
g) Do not know

9. What kind of discomfort or no bowel symptoms have you had repeatedly by consuming foods with wheat or gluten?

a) Hives or rashes that itch
b) Difficulty breathing
c) Swelling of face or lips
d) Fatigue
e) Muscle pain
f) Pain in the joints
g) Lack of Wellbeing
h) Rhinitis
i) Dermatitis
j) Anxiety
k) Confusion

l) Headache
m) Numbness
n) Depression
o) Other (Which one?)
p) None (If you choose this option, please go to question 13)

10. What age were you when you first realized that foods with wheat or gluten caused you the symptoms mentioned in the previous question? _________

a) I do not remember

11. When you eat foods with wheat or gluten, how often the non-intestinal discomfort or symptoms mentioned above occur?

a) Always
b) Most of the times
c) Occasionally

12. After eating foods with wheat or gluten, how long it takes for discomfort referred above to appear?

a) 10 min or less
b) 10 min–1 h
c) 1–2 h
d) In the first 6 h
e) Between 6 and 24 h
f) After 24 h
g) Do not know

13. Have you been diagnosed with any of the following?

a) Irritable bowel syndrome
b) Lactose intolerance
c) Intolerance to food other than lactose (which one?)
d) Allergy (Which one?)
e) Any type of psychiatric disorder?
f) Gastrointestinal cancer
g) Eating disorder (which one?)
h) Autoimmune disease such as diabetes mellitus.
i) Colitis
j) None of the previous diseases

14. Does anyone in your family have been diagnosed with coeliac disease?

a) Yes
b) No
c) Do not know

15. Do you keep a diet free of wheat or gluten?

a) Yes **(go to the next question)**
b) No **(go to question 18)**

16. Who instructs the diet free of wheat or gluten?

a) Nutritionist
b) Dietitian
c) Physician or General practitioner
d) Gastroenterologist
e) Chemist
f) Homeopath
g) Yourself
h) Other, who?

17. What are the main motivations for keeping a diet free of wheat or gluten?

a) Weight control
b) Having a relative with celiac disease or wheat/gluten sensitivity or intolerance
c) The gluten-free products taste better
d) A diet free of wheat or gluten is healthier
e) The symptoms triggered after gluten ingestion
f) Other reason, which one?

(Stop)

18. Do you avoid, as much as possible, wheat- or gluten-based products from your diet?

a) Yes **(Go to the next question)**
b) No **(Stop)**

19. What are the main motivations for avoiding, as much as possible, wheat or gluten from your diet?

a) The symptoms triggered after gluten ingestion
b) Weight control
c) Having a relative with celiac disease or wheat/gluten sensitivity or intolerance
d) The gluten-free products taste better
e) Avoiding wheat or gluten is healthy
f) Other reason, which one?

Thank you very much!

SECTION 2

20. Do you have some discomfort or adverse reaction when consuming a particular food?

a) Yes (Go to next question)
b) No (Please go to question 26)

21. What type of food caused you discomfort or an adverse reaction when you consume it?__________________

a) Do not know

22. What kind of discomfort or gastrointestinal symptoms have you had repeatedly when you consume a particular type of food (s)?

a) Stomachache

b) Diarrhea
c) Vomiting
d) Nausea
e) Reflux
f) Flatulence
g) Bloating
h) Abdominal discomfort
i) Constipation
j) Acidity
k) Other (Which one?)
l) None (Please go to question 24)

23. When you eat foods that cause you discomfort, how often gastrointestinal symptoms mentioned above occur?

a) Always
b) Most of the times
c) Occasionally

24. What kind of no bowel symptoms have you had repeatedly by consuming foods that cause you discomfort?

a) Hives or rashes that itch
b) Trouble breathing
c) Swelling of face or lips
d) Fatigue
e) Muscle pain
f) Joint Pain
g) Lack of Wellbeing
h) Anxiety
i) Confusion
j) Depression
k) Dermatitis
l) Rhinitis
m) Headache
n) Numbness
o) Other (Which one?
p) None (If you choose this option, go to question 26)

25. When you eat foods that cause you discomfort, how often the non-intestinal symptoms mentioned above occur?

a) Always
b) Often
c) Occasionally

26. Have you been diagnosed with any of the following diseases?

a) Lactose intolerance
b) Intolerance to food other than lactose (Which one?)
c) Any type of allergy
d) Any type of psiquiatric disorder
e) Gastrointestinal cancer

f) Eating disorder (Which one?)
g) Autoimmune disease such as diabetes mellitus.
h) Colitis
i) Irritable Bowel Syndrome
j) None of the previous diseases

27. Does anyone in your family have been diagnosed with celiac disease?

a) Yes
b) No
c) Do not know

28. Do you keep a diet free of wheat or gluten?

a) Yes (go to the next question)
b) No (go to question 31)

29. Who instructs the diet free of wheat or gluten?

a) Nutritionist
b) Dietitian
c) Physician or General practitioner
d) Gastroenterologist
e) Chemist
f) Homeopath
g) Yourself
h) Other, who?

30. What are the main motivations for keeping a diet free of wheat or gluten?

a) Weight control
b) Having a relative with celiac disease or wheat/gluten sensitivity or intolerance
c) The gluten-free products taste better
d) A diet free of wheat or gluten is healthier
e) Other reason, which one?

Stop

31. Do you avoid, as much as possible, wheat- or gluten-based products from your diet?

Yes **(go to the next question)**
No **(stop)**

32. What are the main motivations for avoiding, as much as possible, wheat or gluten from your diet?

a) Weight control
b) Having a relative with celiac disease or wheat/gluten sensitivity or intolerance
c) The gluten-free products taste better
d) Avoiding wheat or gluten is healthy
e) Other reason, which one?

Thank you very much!

Spanish version.

1. Presenta algún tipo de malestar o reacción adversa cuando consume productos de trigo, como los elaborados con harina de trigo?

☐ Sí **(pase a la pregunta 2; contesta la sección 1)**

☐ No **(pase a la pregunta 20, contesta la sección 2)**

ID:____ Nombre (opcional):________________________Edad **(importante)**:______________

Femenino ☐ ☐Masculino

Escolaridad (último grado de estudios obtenido):____________________

Contacto (opcional): Email______________________Teléfono___________________________

Por favor señale con una X la respuesta para cada una de las siguientes preguntas

SECCIÓN 1

2. Le han diagnosticado alguna enfermedad relacionada al consumo de trigo o gluten?

☐ Sí **(cuál de las siguientes?)** ☐No **(pase a la pregunta 5)**

☐ Alergia al trigo ☐ Dermatitis herpetiformis ☐Enfermedad celiaca ☐Ataxia por gluten o trigo ☐ Sensibilidad o intolerancia al trigo ☐ Otro Cuál?______________________________

3. Quién realizó el diagnóstico?

☐ Médico general ☐ Gastroenterólogo ☐ Médico homeópata ☐uímico ☐ Nutricionista ☐ Otro Quién?____

4. Hace cuánto tiempo que fue usted diagnosticado con la enfermedad relacionada al consumo de trigo? ____.
(Si no recuerda denos un estimado por favor)

☐ 3 meses o menos ☐ 6 meses o menos ☐ 1 año o menos ☐1–5 años o menos ☐ 6–10 años ☐ más de 10 años

5. Qué tipo de molestias o **síntomas gastrointestinales** ha presentado de forma repetida al consumir alimentos con trigo o gluten?

☐ Dolor de estómago ☐ Diarrea ☐Vómito ☐ Nauseas ☐ Reflujo ☐ Flatulencias ☐Hinchazón de estómago ☐ Malestar abdominal ☐ Estreñimiento ☐ Acidez ☐ Otro Cuál? ☐inguno **(pase a la pregunta 9)**

6. Qué edad aproximadamente tenía cuando se percató de que los alimentos con trigo o gluten le ocasionaban los síntomas mencionados en la pregunta anterior?___________☐o recuerdo

7. Cuando consume alimentos con trigo o gluten, con qué frecuencia se presentan los síntomas gastrointestinales mencionados anteriormente?

☐ Siempre ☐Muchas veces ☐ Ocasionalmente

8. Después de haber comido alimentos con trigo o gluten, Cuánto tiempo demoran en aparecer las molestias gastrointestinales referidas anteriormente?

☐ 10 min o menos ☐ 10 min–1 h ☐ –2 h ☐ n las primeras 6 h ☐ ntre 6 y 24 h
☐ Después de 24 h ☐ No sabe

---

9. Qué tipo de molestias o **síntomas no intestinales** ha presentado de forma repetida al consumir alimentos con trigo o gluten?

☐ Ronchas o sarpullidos que pican ☐ Dificultad para respirar ☐ Hinchazón de cara o labios
☐ Cansancio ☐ Dolor muscular ☐ Dolor en las articulaciones ☐ Falta de bienestar
☐ Rinitis ☐ Dermatitis ☐ Ansiedad ☐ Confusión ☐ Depresión
☐ Dolor de cabeza ☐ Entumecimiento ☐ Otro, Cuál?
☐ Ninguno **(Si elige esta opción pase a la pregunta 13)**

---

10. Qué edad aproximadamente tenía cuando se percató de que los alimentos con trigo o gluten le ocasionaban los síntomas mencionados en la pregunta anterior?________________ ☐ No recuerdo

---

11. Cuando consume alimentos con trigo o gluten, con qué frecuencia se presentan las molestias o síntomas **no intestinales** mencionados anteriormente?

☐ Siempre ☐ Muchas veces ☐ Ocasionalmente

---

12. Después de haber comido alimentos con trigo o gluten, Cuánto tiempo demoran en aparecer las molestias **no intestinales** referidas anteriormente?

☐ 10 min o menos ☐ 10 min–1 h ☐ 1–2 h ☐ n las primeras 6 h ☐ Entre 6 y 24 h
☐ Después de 24 h ☐ No sabe

---

13. Le han diagnosticado alguna de las siguientes enfermedades?

☐ Síndrome de intestino irritable ☐ Intolerancia a la lactosa
☐ Intolerancia a algún otro alimento, Cuál? ________________________________
☐ Alergia Cuál?________________ A☐una enfermedad psiquiátrica Cuál?________________
☐ Cáncer gastrointestinal ☐ Trastorno de conducta alimentaria Cuál?______________
☐ Enfermedad autoinmune como diabetes mellitus. ☐ Colitis ☐ Ninguna de las anteriores

---

14. Alguien en su familia ha sido diagnosticado con enfermedad celiaca?

☐ Sí ☐ No ☐ No sabe

---

15. Lleva usted una dieta libre de trigo o gluten?

☐ Sí **(pase a la siguiente pregunta)** ☐ No **(pase a la pregunta 18)**

16. Quién lo instruye para llevar la dieta libre de gluten?

☐ Nutricionista ☐ Dietista ☐ Medico general ☐ Gastroenterólogo ☐ Químico ☐ Médico homeópata
☐ Usted mismo ☐ Otro, quien?____________________.

17. Cuáles son las principales razones que lo motivan a llevar una dieta libre de gluten?

☐ Control de peso ☐ Tiene un familiar con enfermedad celiaca o intolerancia al trigo
☐ Los productos libres de gluten saben mejor ☐ La dieta libre de gluten es más sana
☐ Los síntomas que le ocasiona Otra razón, cuál?_________________________. **(termina)**

18. Hace usted todo lo posible por no consumir trigo o gluten?

☐ Sí **(pase a la siguiente pregunta)** ☐ No **(termina)**

---

19. Qué lo motiva a hacer todo lo posible por evitar el trigo o gluten en su dieta?

☐ Los síntomas que le ocasiona ☐ Control de peso ☐ Los productos libre de gluten saben mejor
☐ Tiene un familiar con enfermedad celiaca o intolerancia al trigo
☐ Es sano evitar el trigo o gluten de la dieta ☐ Otra razón, cuál?______________________.

¡Muchas Gracias por Participar!

ID:______Nombre (opcional):_______________________Edad (**importante**):______________

Femenino ☐ ☐Masculino

Escolaridad (último grado de estudios obtenido):_____________________.

Contacto (opcional): Email_______________Teléfono______________________________________.________

Por favor señale con una X la respuesta para cada una de las siguientes preguntas

SECCIÓN 2

20. Presenta algún tipo de malestar o reacción adversa cuando consume un alimento en particular **que no sea** elaborado con trigo o harina de trigo?

☐ Sí **(pase a la siguiente pregunta)** ☐No **(pase a la pregunta 26)**

---

21. Que alimento(s) le ocasiona (n) malestar o una reacción adversa cuando lo(s) consume?

☐ No sabe

---

22. Los malestares que presenta al consumir dicho(s) alimento(s), son algunos de los siguientes síntomas gastrointestinales?

☐Dolor de estómago ☐Diarrea ☐Vómito ☐Nausea ☐ Reflujo

☐Flatulencias ☐ Hinchazón de estómago ☐ Malestar abdominal ☐ Estreñimiento

☐Acidez ☐Otro, cuál? _______________________ ☐ Ninguno **(pase a la pregunta 24)**

---

23. Con qué frecuencia se presentan los malestares o síntomas gastrointestinales mencionados anteriormente?

☐ Siempre que consume el alimento ☐La mayoría de las veces que lo consume ☐Ocasionalmente

---

24. Los malestares que presenta al consumir dicho(s) alimento(s), son algunos de los siguientes síntomas no intestinales?

☐ Ronchas o sarpullidos que pican ☐Dificultad para respirar ☐ Hinchazón de cara o labios

☐ Cansancio ☐ Dolor muscular ☐ Dolor en las articulaciones ☐Falta de bienestar

☐ Rinitis ☐ Dermatitis ☐Ansiedad ☐Confusión ☐Depresión ☐Dolor de cabeza

☐ Entumecimiento ☐Otro Cuál?____________________ ☐Ninguno **(pase a la pregunta 26)**

25. con qué frecuencia se presentan las molestias o síntomas **no intestinales** mencionados anteriormente?

☐ Siempre que consume el alimento ☐La mayoría de las veces que lo consume ☐Ocasionalmente

---

26. Le han diagnosticado alguna de las siguientes enfermedades?

☐ Intolerancia a la lactose ☐Intolerancia a otro alimento Cuál? ____________________.
☐ Alergia Cuál?___________. ☐Alguna enfermedad psiquiátrica Cuál?____________.
☐ Cáncer gastrointestinal ☐ Trastorno de conducta alimentaria Cuál?______________.
☐ Diabetes mellitus. ☐Colitis ☐Sindrome de intestino irritable
☐ Ninguna de las anteriores

---

27. Alguien en su familia ha sido diagnosticado con enfermedad celiaca?

☐Sí ☐ No ☐No sabe

---

28. Lleva usted una dieta libre de trigo o gluten?

☐ No **(pase a la pregunta 31)** ☐ Si **(continúe con la siguiente pregunta)**

29. Quién lo instruye para llevar la dieta libre de gluten?

☐ Nutricionista ☐Dietista M☐dico general Gast☐enterólogo Químic☐ Médico h☐meópata
☐ Usted mismo ☐ Otro, quien? ________________________

30. Cuáles son las principales razones que lo motivan a llevar una dieta libre de gluten?

☐ Control de peso ☐ Tiene un familiar con enfermedad celiaca o intolerancia al trigo
☐ Los productos libres de gluten saben mejor ☐La dieta libre de gluten es más sana
☐ Otra razón, cuál?__________________________ **(Termina)**

31. Hace usted todo lo posible por no consumir trigo o gluten?

☐ Si **(pase a la siguiente pregunta)** ☐ No **(Termina)**

32. Qué lo motiva a hacer todo lo posible por evitar el trigo o gluten en su dieta?

☐ Control de peso ☐ Tiene un familiar con enfermedad celiaca o intolerancia al trigo
☐ Es sano evitar los productos con trigo o gluten ☐Los productos libres de gluten saben mejor
Otra razón, cuál?______________________.

Muchas Gracias por Participar!

S2. Tables and Figures

Table S1. Comparison between self-reported gluten sensitivity (SR-GS) and recurrent adverse reactions to foods different from wheat/gluten groups.

Variable *	Recurrent Adverse Reactions				Odds Ratio (95% CI)
	Wheat/Gluten (*N* = 92) (SR-GS)		Other Foods (*N* = 102)		
	-------	*n*	-------	*n*	
Mean age in years (range) **	33.5 (18–74)	-------	33.4 (18–81)	-------	-------
Gender (female/male) (%)	53.8/46.2	49/42	68.6/30.4	71/31	0.51 (0.28–0.92)
Irritable bowel syndrome (%)	14.3	13	11.8	12	1.25 (0.54–2.90)
Food intolerance (%)	3.3	3	2.9	3	1.12 (0.22–5.72)
Allergy (%)	11.0	10	12.7	13	0.85 (0.35–2.03)
Psychiatric disease (%)	0	0	3.9	4	-------
Gastrointestinal cancer (%)	1.1	1	0	0	-------
Eating disorders (%)	4.4	4	2.9	3	1.52 (0.33–6.97)
Autoinmune disease (%)	2.2	2	2.9	3	0.74 (0.12–4.54)
Colitis (%)	4.4	4	1.0	1	4.64 (0.51–42.33)
Lactose intolerance (%)	6.6	6	5.9	6	1.13 (0.35–3.63)

* Self-reported physician-diagnosed diseases were considered for analysis; ** Age comparison by student's *t*-test ($p > 0.05$).

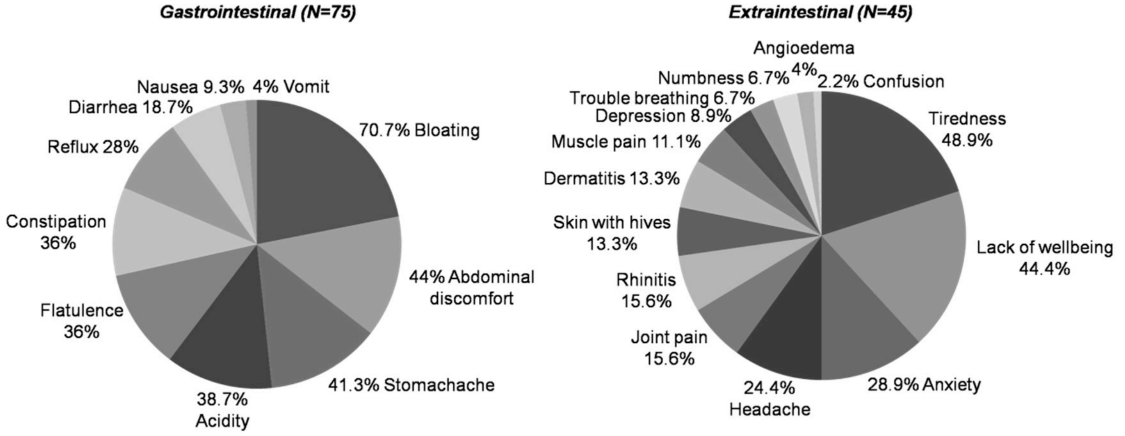

Figure S1. Self-reported symptoms in the SR-NCGS group.

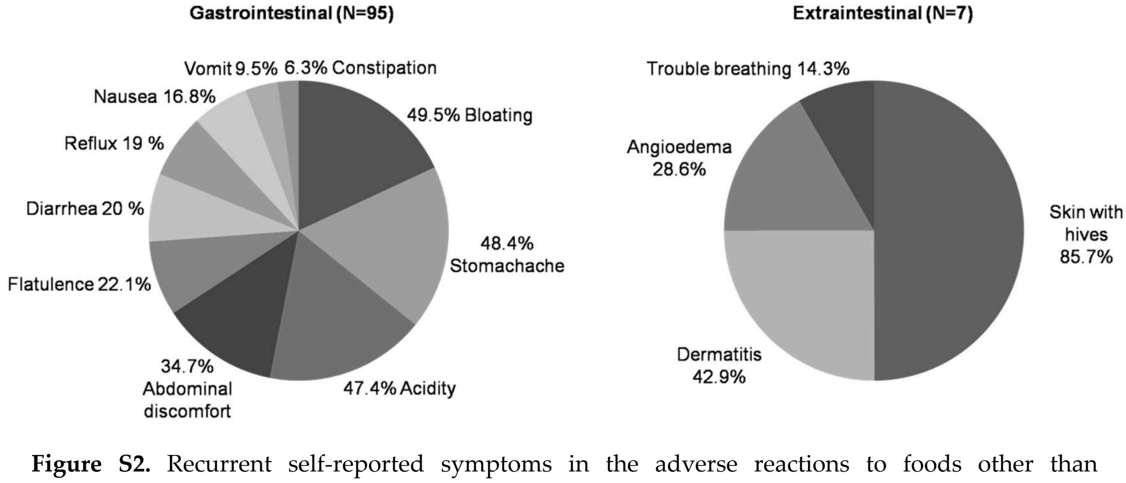

Figure S2. Recurrent self-reported symptoms in the adverse reactions to foods other than wheat/gluten group.

Table S2. Symptoms comparison between identified SR-GS and recurrent adverse reactions to foods other than wheat/gluten cases.

Gastrointestinal Symptoms	Recurrent Adverse Reactions to Wheat/Gluten (SR-GS) (*N* = 92)		Recurrent Adverse Reactions to Foods Other Than Wheat/Gluten (*N* = 102)		Odds Ratio (95% CI)
	%	*n*	%	*n*	
Bloating	70.1	61	49.5	47	2.3 (1.3–4.1)
Abdominal discomfort	47.1	41	34.7	33	1.7 (0.9–3.0)
Stomachache	46	40	48.4	46	0.9 (0.5–1.7)
Acidity	36.8	32	47.4	45	0.7 (0.4–1.2)
Constipation	34.5	30	6.3	6	7.7 (3.0–19.7)
Flatulence	33.3	29	22.1	21	1.8 (0.9–3.4)
Reflux	28.7	25	19	18	1.7 (0.9–3.5)
Diarrhea	25.3	22	20	19	1.4 (0.7–2.7)
Nausea	14.5	13	16.8	16	0.9 (0.4–2.0)
Vomit	10.3	9	9.5	9	1.1 (0.4–3.0)
**Extraintestinal Symptoms**	**Recurrent Adverse Reactions to Wheat/Gluten (SR-GS) (*N* = 92)**		**Recurrent Adverse Reactions to Foods Other Than Wheat/Gluten (*N* = 102)**		**Odds Ratio (95% CI)**
	%	*n*	%	*n*	
Tiredness	55.6	25	0	0	----------
Lack of wellbeing	44.4	20	0	0	----------
Anxiety	28.9	13	0	0	----------
Skin with hives	26.7	12	85.7	6	2.4 (0.9–6.7)
Headache	26.7	12	0	0	---------
Dermatitis	15.6	7	42.9	3	2.7 (0.7–10.8)
Rhinitis	15.6	7	0	0	----------
Angioedema	13.3	6	28.6	2	3.5 (0.7–17.7)
Muscle pain	13.3	6	0	0	----------
Joint pain	13.3	6	0	0	----------
Depression	13.3	6	0	0	----------
Trouble breathing	8.9	4	14.3	1	4.6 (0.5–41.8)
Numbness	6.7	3	0	0	----------
Confusion	2.2	1	0	0	----------

Table S3. Motivations for following a GFD and avoiding wheat/gluten-containing foods.

Motivations	Comparisons between SR-GS and Non-SR-GS Groups: GFD			Avoiding Wheat/Gluten		
	SR-GS	Non-SR-GS	*p* Value	SR-GS	Non-SR-GS	*p* Value
Weight control	6 (27.3%)	29 (57.7%)	0.037	4 (8.7%)	95 (63.8%)	<0.001
Relative with CD	2 (9.1%)	2 (3.6%)	0.321	0	6 (4.0%)	--------
Gluten-free products taste better	0	1 (1.8%)	--------	0	2	--------
GFD is healthier	3 (13.6%)	22 (40.0%)	0.021	-------	--------	--------
Avoiding gluten is healthy	-------	-------	--------	2 (4.3%)	31 (20.8%)	0.005

Table S4. Motivations for following a GFD and who instructs the GFD in the SR-GS group stratified by gender.

Motivations for Following a GFD						
**Gender**	***N***	**Weight Control**	**Relative with CD**	**Gluten-Free Products Taste Better**	**GFD is Healthier**	**Symptoms Only**
Female	15	5 (33.3%)	2 (13.3%)	0	1 (6.7%)	7 (46.7%)
Male	7	1 (14.3%)	0	0	2 (28.6%)	4 (57.1%)
**Who Instructs the GFD**						
**Gender**	***N***	**Dietitian**	**Yourself**	**Physician**	**Other**	**----------**
Female	15	6 (40%)	8 (53.3%)	1 (6.7%)	0	----------
Male	7	3 (42.9%)	4 (57.1%)	0	0	----------
